# Vein of Galen Malformation Masquerading as Neonatal Respiratory Distress

**DOI:** 10.7759/cureus.109082

**Published:** 2026-05-18

**Authors:** Hadi Fakih, Hussein A Hussein, Fatima Fakih

**Affiliations:** 1 Pediatrics, Sheikh Ragheb Harb University Hospital, Nabatieh, LBN; 2 Pediatrics, Faculty of Medical Sciences, Lebanese University, Beirut, LBN; 3 Neonatology, Faculty of Medicine, Lebanese University, Beirut, LBN

**Keywords:** cranial ultrasound, high-output heart failure, hydrocephalus, macrocephaly, neonatal respiratory distress, vein of galen malformation

## Abstract

Vein of Galen malformation (VoGM) is a rare congenital vascular anomaly. It represents a well-recognized extracardiac cause of high-output cardiac failure in the neonatal period. We report a case of a 3.5-month-old boy who presented for routine vaccination and was incidentally found to have macrocephaly (head circumference 44.5 cm, >99th percentile) with early sun-setting eyes. He was otherwise well-appearing with a normal neurological examination. Bedside cranial ultrasound revealed bilateral lateral and third ventricular dilation with a cystic mass compressing the aqueduct, and brain MRI confirmed a dilated cystic malformation of the vein of Galen as the cause of obstructive hydrocephalus. His past medical history was significant for severe neonatal respiratory distress complicated by pulmonary hypertension and congestive heart failure, initially attributed to neonatal pneumonia. This case highlights that VoGM should be considered in the differential diagnosis of neonatal persistent pulmonary hypertension (PPHN) and high-output heart failure, even when a concurrent respiratory illness is present, and emphasizes the value of bedside cranial Doppler ultrasound and thorough physical examination including fontanel auscultation.

## Introduction

Hydrocephalus in infancy can result from either communicating or obstructive etiologies. Obstructive hydrocephalus in the first year of life is most commonly caused by aqueductal stenosis, Chiari malformations, Dandy-Walker malformation, or space-occupying lesions such as tumors or vascular malformations [1]. Among vascular causes, vein of Galen malformation (VoGM) is a rare but important etiology. A systematic review and meta-analysis reported an incidence of roughly one in 25,000 live births, with a pooled mortality rate of 40.6% in untreated patients [2].

VoGM results from an abnormal arteriovenous shunt that develops between the primitive choroidal arteries and the median prosencephalic vein of Markowski during the sixth to 11th weeks of gestation [3]. Persistent high-flow shunting prevents normal regression of this embryonic vein, leading to progressive dilation of the venous structure [4]. The clinical presentation varies with age: neonates typically present with high-output congestive heart failure due to increased shunting after birth [5], while infants beyond the neonatal period more commonly present with hydrocephalus, macrocephaly, or seizures [6]. Hydrocephalus in VoGM results from both direct aqueductal obstruction by the dilated venous pouch and impaired CSF absorption due to venous hypertension [2]. This case report describes an infant whose VoGM presented initially as severe neonatal respiratory distress and heart failure, misattributed to pneumonia and was only discovered months later when routine examination revealed progressive macrocephaly.

## Case presentation

A 3.5-month-old male infant presented to the pediatric clinic for routine vaccination. He was born at 39 weeks of gestation via programmed cesarean section to a 33-year-old mother (G3P3A0). Antenatal follow-up was unremarkable except for maternal depression, for which the mother was maintained on fluoxetine 10 mg daily throughout pregnancy.

Birth weight was 3,200 g, and Apgar scores were 6 at one minute and 8 at five minutes. Head circumference at birth was 38 cm (>99th percentile for boys, WHO growth charts), indicating macrocephaly present since birth.

In the past medical history, the neonatal period was complicated by severe respiratory distress within the first 24 hours of life, initially treated at another facility for severe neonatal pneumonia. Due to clinical deterioration requiring high-frequency oscillatory ventilation (HFOV) and inhaled nitric oxide (iNO), he was transferred to our tertiary care center (Sheikh Ragheb Harb University Hospital) at five days of life.

On arrival, echocardiography revealed biventricular enlargement, reduced systolic function, and elevated pulmonary artery pressures consistent with persistent pulmonary hypertension (PPHN) and high-output congestive heart failure. He was treated with captopril, furosemide, spironolactone, amiodarone, and sildenafil. Serial echocardiograms demonstrated gradual improvement, and by two months of age, his regimen was weaned to furosemide monotherapy. No cranial imaging was performed during the neonatal admission.

**Figure 1:**
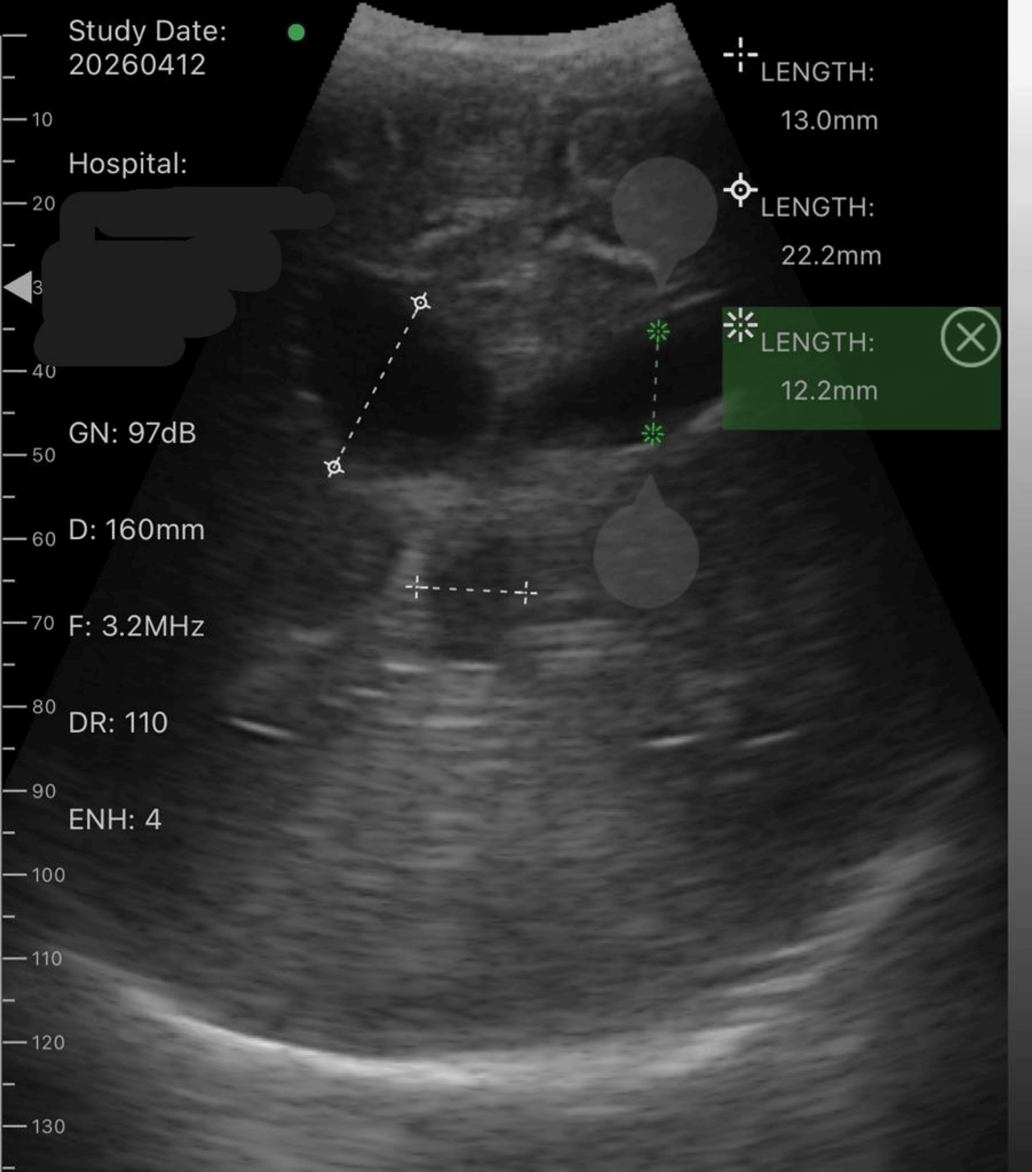
Bedside cranial ultrasound images. Coronal view demonstrating dilated lateral ventricles.

**Figure 2:**
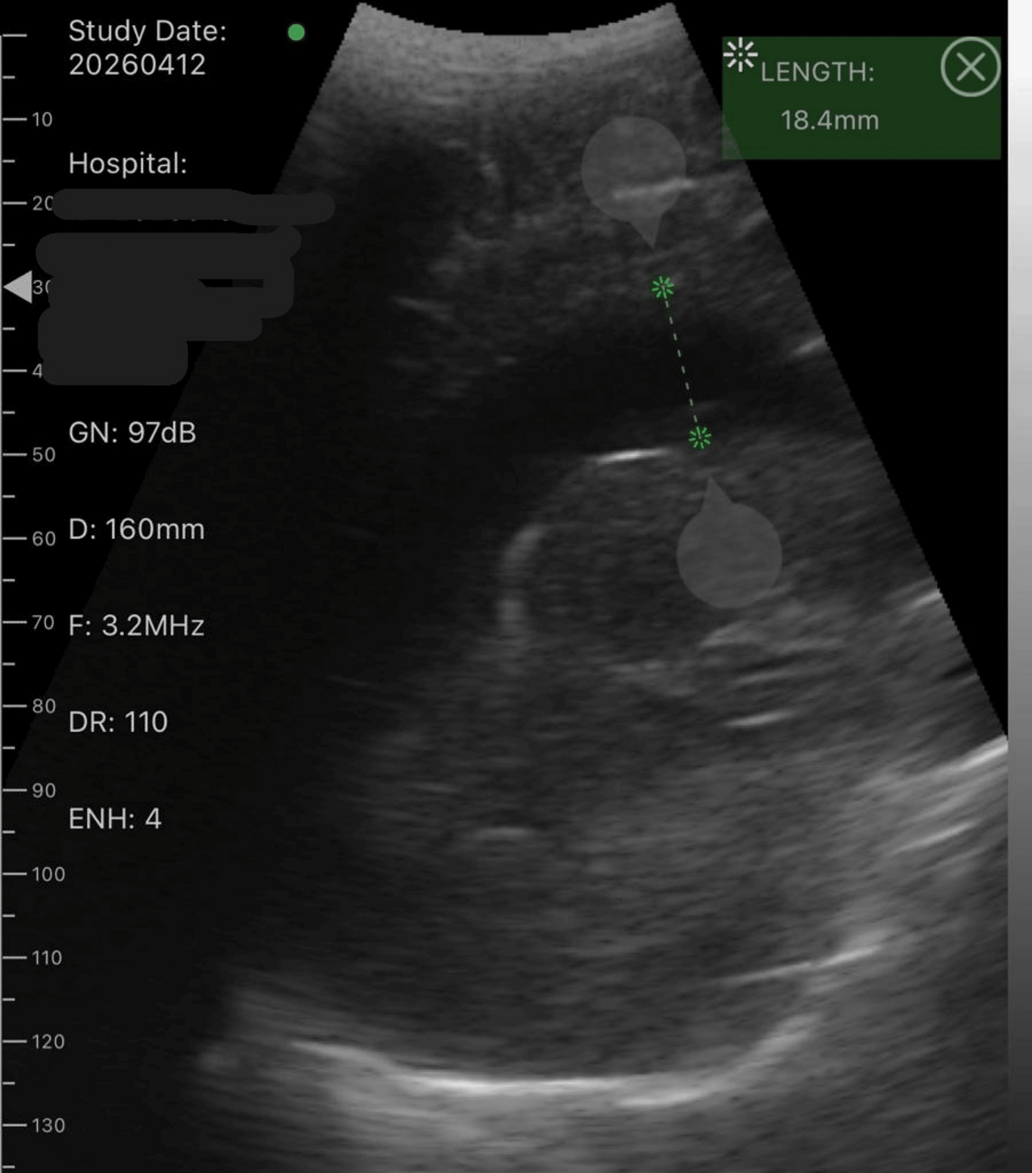
Sagittal view showing dilated lateral and third ventricles.

**Figure 3:**
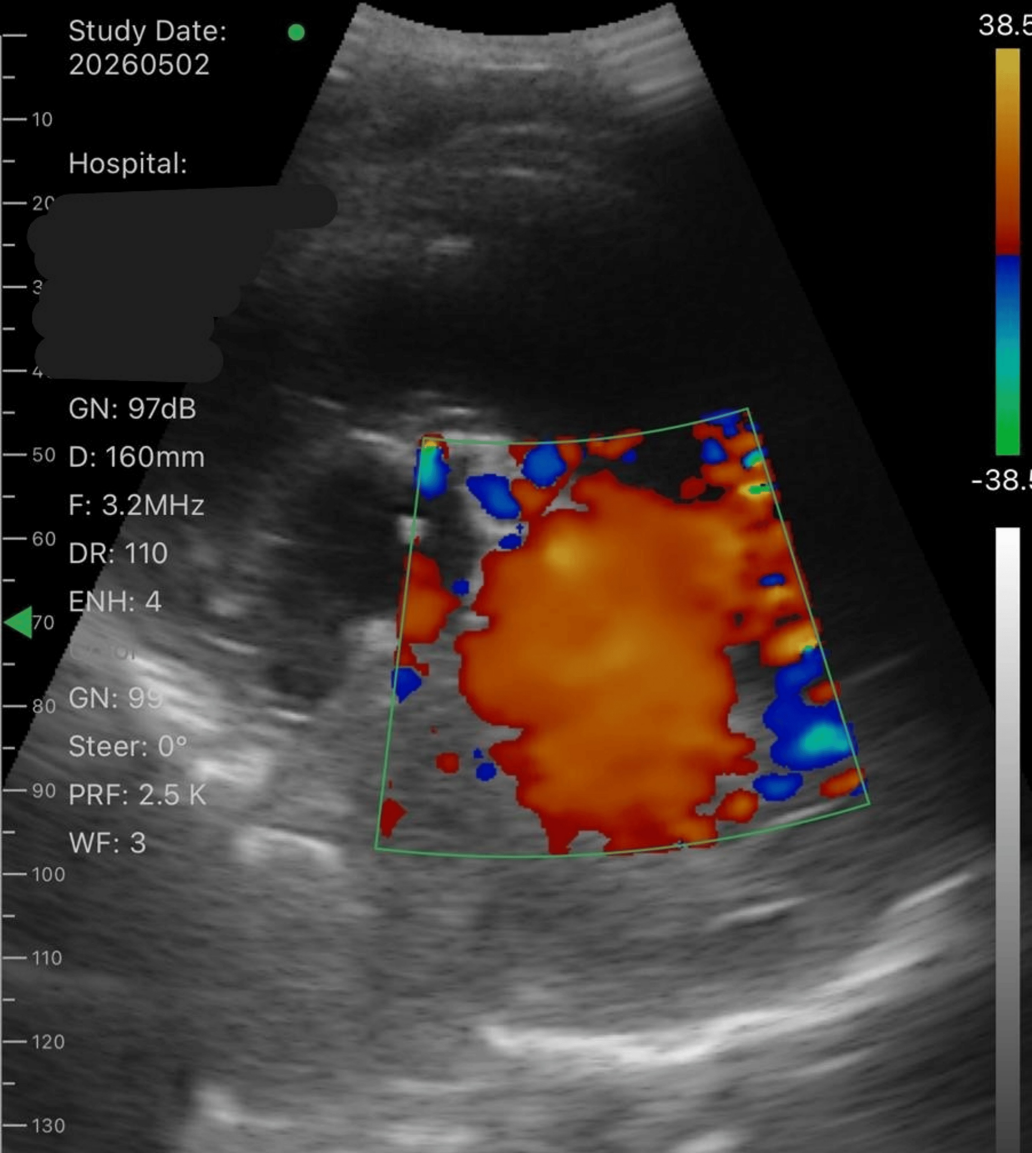
Doppler ultrasound revealing turbulent flow within a cystic structure posterior to the third ventricle, consistent with a vascular malformation.

At the 3.5-month well-child visit, the infant was noted to have macrocephaly with a head circumference of 44.5 cm (>99th percentile). Physical examination revealed a well-appearing, afebrile infant with early sun-setting eyes. The anterior fontanel was open and bulging. Neurological examination was normal. On auscultation of the fontanel and cranium, a soft bruit was heard. Bedside cranial ultrasound revealed bilateral lateral and third ventricular dilation with a hypoechoic cystic mass posterior to the third ventricle causing aqueductal compression with turbulent flow on Doppler interrogation (Figures 1-3).

**Figure 4:**
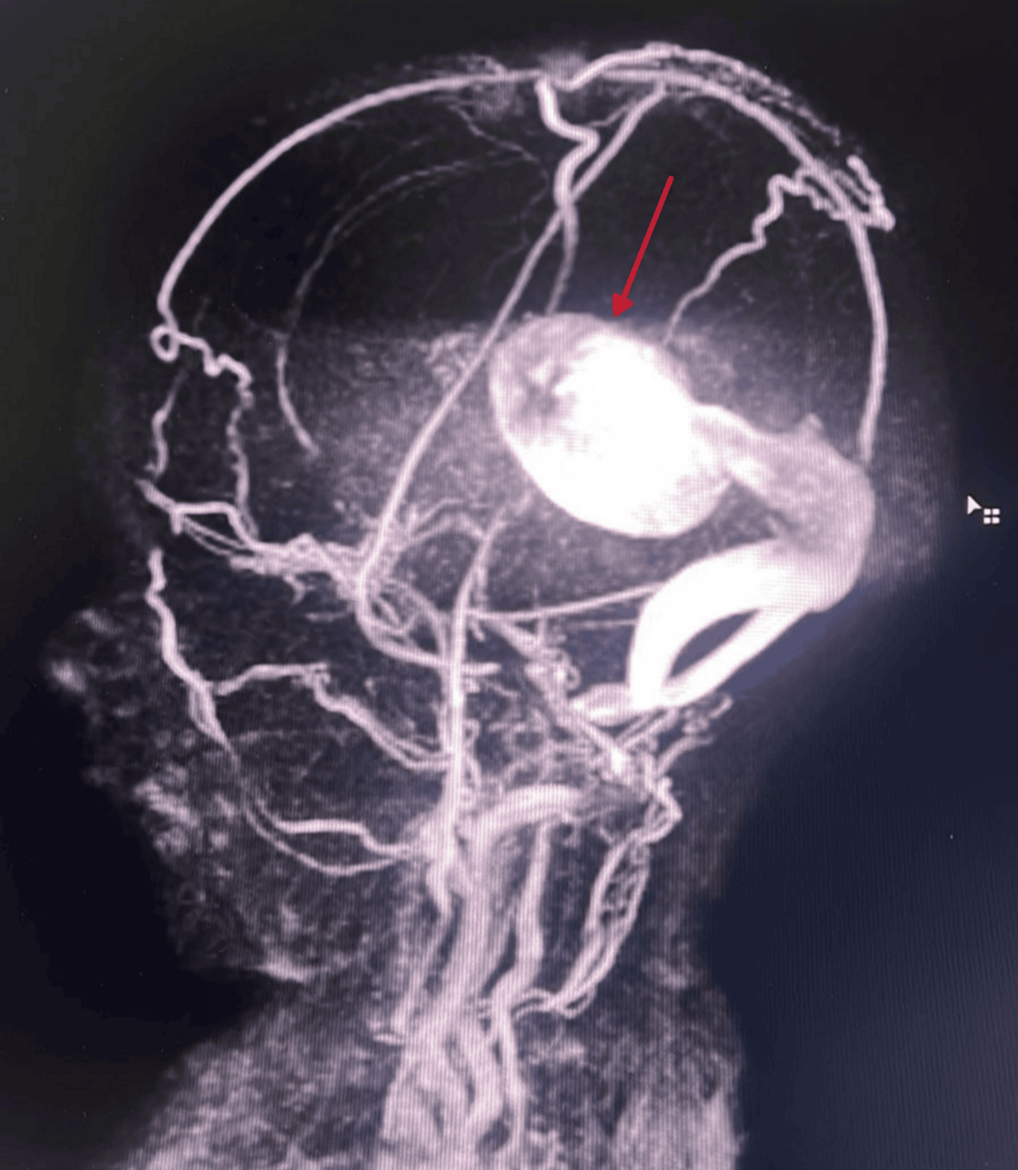
Magnetic resonance venography (MRV) of the brain showing the vein of Galen malformation. The arrow indicates the dilated venous pouch with associated arterial feeders and venous drainage pattern.

Brain MRI and magnetic resonance venography (MRV) confirmed a dilated, cystic malformation of the vein of Galen measuring approximately 2.1 cm, with multiple arterial feeders from the posterior cerebral and anterior choroidal arteries, causing obstructive hydrocephalus (Figure 4).

The infant was urgently referred to a pediatric neurovascular center for evaluation for endovascular embolization.

## Discussion

The differential diagnosis of neonatal respiratory distress is broad, encompassing pulmonary, cardiac, infectious, structural, and metabolic causes [7]. In our patient, the initial diagnosis of neonatal pneumonia with secondary PPHN was reasonable but ultimately incomplete. Maternal fluoxetine use during pregnancy may have contributed to PPHN, as selective serotonin reuptake inhibitors (SSRIs) are associated with a two- to threefold increased risk [8,9]. However, the persistence of heart failure should have raised suspicion for an underlying extracardiac shunt such as VoGM, the most common cause of high-output cardiac failure in newborns [5]. The pathophysiological mechanism involves reduced systemic vascular resistance after birth, increased shunt fraction, pulmonary overcirculation, and biventricular failure [4].

VoGM results from persistent embryonic shunting between choroidal arteries and the median prosencephalic vein of Markowski [3]. Anatomically, it is classified into choroidal (high-flow, neonatal heart failure) and mural (lower-flow, infantile hydrocephalus) types [3]. A validated clinical staging system (Mortazavi classification) grades patients from I (no compromise) to V (irreversible multiorgan failure) [10]. Our patient presented with neonatal heart failure requiring intensive care without documented cerebral injury (Grade III), then progressed to hydrocephalus with macrocephaly by 3.5 months while remaining neurologically normal. The diagnosis was delayed due to attribution bias, potential confounding by maternal SSRI use, lack of cranial auscultation for bruit (present in 60%-80% of cases [4]), and absence of cranial imaging during the neonatal admission. A bedside cranial ultrasound with Doppler is a rapid, non-invasive diagnostic tool that can readily identify VoGM and assess ventricular size and parenchymal injury. Definitive diagnosis requires MRI/MRV and digital subtraction angiography. Endovascular embolization is the current standard of care, with staged procedures to reduce shunting gradually [6]. Prognosis depends on presentation severity: untreated symptomatic VoGM has 50%-100% mortality, while modern endovascular techniques have reduced mortality to 4%-40% [6]. Poor prognostic factors include neonatal heart failure, choroidal type, pre-existing brain injury, and delayed diagnosis [2-4,6]. The "vascular steal" phenomenon, causing chronic cerebral hypoperfusion from shunting, can lead to progressive brain injury, emphasizing the need for early diagnosis and treatment [4].

## Conclusions

VoGM is a rare but critical entity that must remain in the differential diagnosis of neonatal PPHN and high-output heart failure, including when maternal SSRI use provides an alternative explanation. Bedside cranial Doppler ultrasound is a rapid, non-invasive diagnostic tool that should be performed early in any infant with unexplained macrocephaly or concerning cardiac findings. Auscultation of the fontanel and cranium for a bruit remains a simple but potentially life-saving maneuver. Early diagnosis enables timely referral for endovascular embolization, improving both cardiac and neurological outcomes.
